# Autistic Traits Affect Reward Anticipation but not Reception

**DOI:** 10.1038/s41598-020-65345-x

**Published:** 2020-05-21

**Authors:** Magdalena Matyjek, Mareike Bayer, Isabel Dziobek

**Affiliations:** 1 Berlin School of Mind and Brain, Humboldt-Universität zu Berlin, Berlin, Germany; 2 Department of Psychology, Humboldt-Universität zu Berlin, Berlin, Germany

**Keywords:** Social neuroscience, Neurophysiology, Human behaviour

## Abstract

Autism spectrum conditions (ASC) have been linked to aberrant reward processing, but it remains unclear whether it is a general dysfunction or limited to social stimuli, and whether it affects both phases of reward processing, namely anticipation and reception. We used event-related brain potentials and a population-based approach to investigate reward anticipation and reception to socially relevant (i.e., picture of experimenter’s face showing approval/disapproval) and monetary rewards in 51 neurotypical individuals with varying levels of autistic traits. Higher autistic traits were associated with enhanced reward anticipation across reward types in the early anticipation phase (triggered by incentive cues), but not in the late anticipation phase (directly before reward reception), as reflected by the CNV component. The P3 component in response to reward reception showed a general increase for monetary outcomes, which was not modulated by autistic traits. These results suggest that higher autistic traits are related to enhanced reward anticipation, but do not modulate reward reception. No interaction between reward types and autistic traits was observed. We propose that the relevance of social rewards had higher reward value than commonly used pictures of strangers, which specifically normalised responses for individuals with high autistic traits.

## Introduction

Autism Spectrum Conditions (ASC) are characterised by persistent deficits in communication and social interactions^1^. The *social motivation* account of ASC^2,3^ suggests a decreased sensitivity and responsivity to social incentives (e.g. smiles) and consequently a diminished motivation for social interaction in individuals with ASC. Abnormal activation of the brain’s reward system might cause children with ASC to appreciate and enjoy social stimuli less, which normally motivate typically developing children to interact^4^. This lack of social motivation might lead to withdrawal from interactive situations, and therefore to deprivation of social and emotional input. In turn, insufficient exposure to interacting social environments might impair acquisition and development of communicative and social skills^5^. The social motivation account proposes that social stimuli have lower rewarding power for individuals with ASC^6^. However, this line of research was challenged by work showing general impairments in reward processing, which are not limited to the social domain^7,8^, and work showing no differences or even enhanced reward responsiveness in ASC^9–11^. Thus, so far it remains unclear whether a possible reward dysfunction in ASC is limited to the social domain, or manifests more generally.

One way to address this discrepancy in the literature is to use a population-based approach, in which individuals with ASC represent extreme values on the continuous distribution of autistic traits in the population^12^. Autistic traits are a set of personality characteristics that reflect the phenotypic expression of the genetic liability of autism^13^, and can be measured with questionnaires, like the Autism Spectrum Quotient (AQ^14^). Both higher levels of autistic traits in a sub-clinical population and increased severity of autism symptomatology in ASC have been linked with abnormal reward processing^15–17^. Importantly, population-based studies inform psychopathological approaches (contrasting ASC-diagnosed vs. control subjects) by exploring reward processing within neurotypical groups. Neglecting heterogeneity of responsiveness to rewards in control groups can contribute to inconsistencies in the literature (and stress the need for careful assessment of autistic traits in subclinical individuals).

Another notable factor when trying to explain inconsistencies in the literature is the nature of social stimuli used across studies. Even though there is evidence that social familiarity normalises face processing in ASC^18^, most studies have utilised pictures of strangers as social feedback, which may reduce the reward value of these stimuli, and thus decrease social motivation. Furthermore, social anxiety, which is a common comorbidity of autism^19^, may also influence reward responsiveness to social stimuli, as it is known to modulate face processing^20^. Research concerned with reward processing in social contexts should therefore account for social relevance of the presented faces and face-processing biases linked to social anxiety.

Finally, reward processing comprises two distinctive phases, namely anticipation and reception^21^, which can be differentially affected in individuals with reward processing dysfunction, e.g. in addiction^22^. Anticipation is the motivational, appetitive phase in which subjects seek and await reward. Usually the subsequent reception of reward is related to “liking” the outcome and experiencing pleasure. Research using electroencephalography (EEG) and neuroimaging is sparse and inconclusive about the pattern of possible reward processing impairments in autism, with studies reporting reduced processing only in anticipation of rewards or in both phases (e.g.^15,23^ and^24,25^, respectively). Moreover, some studies^8,26–29^ have targeted only one of the phases, thus providing only a partial picture of reward responsiveness.

To investigate anticipation and appreciation of rewards separately, a high temporal resolution technic is required. Event-related potentials (ERPs) – brain components extracted from the EEG signal – allow measuring brain responses with millisecond resolution, which is lacking in hemodynamic neuroimaging methods. ERPs have been effective in dissociating anticipation and reception in response to rewards in autism research, revealing atypical responses to anticipation^8,16,24,30^ or reception^17,24,27^ of rewards. Anticipatory brain processes are reflected in the EEG signal as late negative potentials elicited at the central-parietal sites and associated with expectation of an upcoming stimulus, like the Contingent Negativity Variation (CNV^31^). The CNV amplitude has been reported to be modulated by motivation, effort, and the anticipation of affective or motivationally salient stimuli^32^. More elaborate, cognitive and affective stimulus processing is reflected in the P3 – a positive, centro-parietal component related to allocation of attentional resources. In the context of reward processing, it is believed to reflect the motivational significance of a reward^33^.

In this study we investigated the modulatory effects of autistic traits on reward anticipation and reception in social and non-social domains. We used a modified version of the cued incentive delay task^34^, with symbolic incentive cues indicating the type of outcome in a given trial. In order to achieve a more natural, real-life setting and to normalise possible differences in processing irrelevant faces linked to autistic traits, we used pictures of the main experimenter, i.e., a socially relevant interaction partner, as social reward stimuli. All participants met the experimenter for the first time on the day of the study and spent the same amount of time with her during the EEG preparations and verbal instructions for the tasks. This standardised exposure assured that the experimenter’s familiarity was naturally built in the interaction. The shared social context and the importance of the experimenter in this situation were designed to make her face more socially relevant. In line with the social motivation account, we hypothesised that participants with higher levels of autistic traits (as measured with AQ) would display attenuated amplitudes of the CNV while anticipating social rewards (possibly also familiar faces^35^), than participants with low levels of autistic traits^16^. We hypothesised that autistic traits would modulate the reward reception measured with the P3 in the social, but not in the non-social condition^17,29^.

To our knowledge no study has focused on the time course of anticipation, i.e. modulation of these responses in relation to early or late stages of anticipation in relation to autistic traits, which we targeted for an exploratory analysis in this study^36^.

## Methods

### Participants

55 volunteers participated in the study; the data sets of 4 participants were excluded due to poor EEG signal quality (1), inefficient language proficiency (1) and unreliable questionnaire data, i.e. inconsistent answer patterns (2). The remaining sample (26 women, 25 men) had a mean age of 27.8 years (*SD* = 4.6). Forty-eight participants were right-handed^37^; all had normal or corrected-to-normal vision. All participants were recruited via flyers published on eBay Kleinanzeigen (a popular advertising platform in Germany) and distributed at Berlin’s university campuses. Inclusion criteria were age (18–50), proficiency in German, no history of psychological, neurological or psychiatric disorders in the last 6 months (including medication), and no past diagnosis of such. After the experiment, the aims of the study and the focus on autistic traits were revealed to the participants in a debriefing conversation. One participant reported then self-suspected autism with two diagnostic investigations in specialised autism diagnosis centres; both of which did not confirm a diagnosis of ASC. Participants were compensated 8 Euro per hour plus additional 4 Euro as a monetary reward earned during the task (for details, see section Stimuli and task), which resulted in a total reimbursement of 20 Euro. All participants provided written informed consent; the study was approved by the ethics committee of the Faculty of Psychology of the Humboldt-Universität zu Berlin and was conducted in accordance with the Declaration of Helsinki.

### Stimuli and task

Participants performed a cued incentive delay task, in which they had to guess the colour of a card drawn from a deck. For each correct response they received a reward – either a picture of a smiling face of the experimenter (a natural, relevant social reward), a monetary reward, or both combined. Participants were instructed about the task both in writing and verbally.

**Figure 1:**
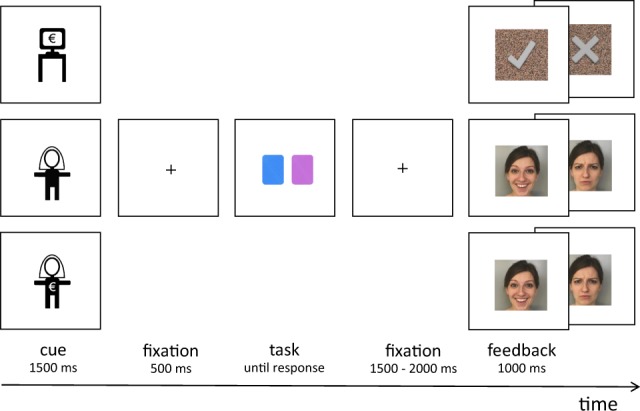
The cued incentive delay task with three conditions (from the top): monetary, combined (social + monetary), social.

For an overview of the task, see Fig. 1. In each trial, a cue (3 ×2.5 visual angles) indicating the reward type in a given trial was presented at the centre of the screen for 1000 ms. The cue was followed by a small white fixation cross (0.7 ×0.7 of visual angle) displayed centrally for 500 ms. Then, a blue and a purple card were displayed on both sides of the screen until the participant’s response. Participants were instructed to make a guess about the colour of the next card drawn from a deck consisting of blue and purple cards. Participants were required to press one of two response buttons indicating their choice (for the left and right card, respectively). The location (right or left) of the blue and the purple cards on the screen was random. There was no time constraint for the response. During the pre-feedback waiting period a fixation cross was presented for 1500 to 2000 ms (jittered across trials). A feedback stimulus (matching the incentive type indicated by the cue) was presented after the pre-feedback anticipation phase for 1000 ms.

Three incentive conditions were introduced: social reward (S), monetary reward (M), and combined social + monetary reward (SM). Correct and incorrect responses in both the S and the SM condition were rewarded with a picture of a happy/approving or a disapproving face of the main experimenter, respectively. Participants were informed in the instructions that in the SM condition they received 5 cents as well as a smile and were aware of the respective conditions when engaging with the trials given the incentive cues that were provided. In the M condition, a symbol of a grey tick served as an indicator of the reward; an incorrect guess was followed by a grey cross. Both symbols were displayed on a background made of scrambled pixels of the S rewarding feedback picture (the happy/approving face). All feedback stimuli were equal in size (3 ×3° of visual angle) and luminance. The feedback stimuli were presented in the centre of the screen for 1000 ms. Participants were instructed that in the M and the SM conditions each correct guess was rewarded with 5 cents. The current balance was displayed after each block. Participants were told that the study focused on decision making in game situations. However, in reality, participants’ decisions were not influencing the feedback valence (to exclude the possible advantage of individuals with low intensity of autistic traits in learning an implicit rule^38^).

Altogether, 9 experimental blocks were presented pseudo-randomly. The first three blocks were of 10:18 proportion of reward to no-reward feedback. In the next three blocks the proportion changed to 14:14, and in the last three to 18:10. This grouping was designed to elicit a sense of agency and performance improvement over time in the participants (and eliminate possible frustration). Each group of three blocks consisted of one block for each incentive type (S, M, and SM; each block included only one type). The first 3 blocks were presented in random order. The next 2 groups of 3 blocks were presented in the same order as in the first 3 blocks. Each block consisted of 28 trials, resulting in 252 trials in total.

### Procedure

After signing the consent form, participants were prepared for the EEG recording, during which time the experimenter maintained a light social conversation detailed by an interaction script. This was administered to achieve a natural familiarisation with the experimenter, with whom all participants spent the same amount of time and were exposed to various viewing angles of her face and her facial expressions. To emphasise the shared social context, the experimenter also pointed out that this study was her project and she cared for the participants’ performance in the task. Then, participants were seated in a dimmed, electrically shielded room at a distance of 70 cm from a 19-inch computer screen. Participants were asked to place their chin and forehead on a head-rest in order to restrain movements.

After the recording, participants answered a number of debriefing questions and completed questionnaires displayed on a computer screen (see Questionnaires and debriefing questions). Then they were debriefed and informed about the real purpose of the study.

### EEG data acquisition and pre-processing

The continuous scalp EEG was recorded from 64 silver/silver-chloride active electrodes (Biosemi Active Two) with a 512 Hz sampling rate. The electrodes were secured in an elastic cap according to the extended 10–20 international electrode placement system. Signals were referenced online to the CMS-DRL ground loop, which drives the average potential as close as possible to the amplifier zero. The electrode offsets were kept within the range of +/− 20 microvolts. The horizontal and vertical electro-oculograms were acquired from four external electrodes, placed at the outer canthi and below both eyes. Two electrodes were additionally positioned on the left and right mastoids. The signals were filtered online with a 100 Hz low-pass and 0.01 Hz high-pass filter.

Offline, each signal was re-referenced to the average of all signals. Data were filtered with a low-pass filter of 40 Hz (slope 8 dB/oct). Channels with poor signals were interpolated using spherical splines of order 4 (0.1% of all channels). Continuous data were segmented into epochs ranging from −100 ms before to 7000 ms after the cue onset. All segments were referred to a 100 ms pre-cue baseline. An independent component analysis algorithm (restricted fast ICA) was used to identify and remove blinks and eye movements. Each segment was then further divided into three sub-segments related to the presentation of the incentive cue, the anticipation phase before the feedback, and the feedback. Respectively, the sub-segments were ranging from −100 ms before to 1500 ms after the cue onset; from −600 ms before feedback onset to feedback onset; and from −100 ms to 1000 ms after the feedback stimuli onset. We time-locked anticipatory responses to multiple events (cue and pre-feedback) to allow for a comparison of the CNV amplitudes across time, investigating how anticipation is built and sustained across the paradigm. A semi-automatic artifact rejection was applied to all epochs, excluding activations exceeding +/− 100 microvolts or voltage steps larger than 100 microvolts. This led to rejection of 1.3% of trials for cue signals, 2.1% of trials for pre-feedback, and 0.8% for feedback signals. Across participants an average of 83 artifact-free trials was obtained in each condition for the cue responses (SM: *SD* = 3.9, M: *SD* = 2.1, S: *SD* = 2.7). Average number of pre-feedback segments was 82 in S and SM, and 83 in M (SM: *SD* = 4.8, M: *SD* = 2.5, S: *SD* = 4.3). For the feedback responses the average number of trials was 42 in each condition and reward/no-reward outcome (*SD*s for reward SM, S, and M, and no-reward SM, S, and M, respectively: 1.8, 1, 0.8, 1.9, 1.3, 1.2). Number of artifact-free segments for cues, pre-feedback and feedback were not significantly different between conditions in each phase (all *F*s < 1.15, *p*s > 0.32). Furthermore, the number of cue and pre-feedback segments did not differ significantly (*t* = 1.92, *p* = 0.06). Finally, epochs were averaged per subject, condition (S, M, SM) and separately for cues, pre-feedback, and feedback. All offline pre-processing steps were performed using BrainVision Analyzer (Brain Products GmbH, Munich, Germany).

### Questionnaires and debriefing questions

All participants completed an online version of the Autism Spectrum Quotient (AQ^14^) prior to the experiment. The AQ is a self-administered questionnaire assessing the degree of traits associated with autism in neurotypical individuals consisting of 50 items. In the original study evaluating the AQ, the control group scored on average 16.4 and the autism group 35.8 (Baron-Cohen *et al*., 2001). In this study participants’ scores ranged from 6 to 35, with the mean of 18.3 (*SD* = 7.3). Females and males did not differ in their mean AQ score [*t*(48.92) = −1.03, *p* = 0.31].

After EEG recording, participants completed the Behavioural Inhibition and Approach Systems Scales (BIS/BAS^39^), the Liebowitz Social Anxiety Scale self-reported (LSAS-SR^40^) and the Edinburgh Handedness Inventory^37^.

The BIS/BAS questionnaire assesses two motivational systems, the behavioural activation system (BAS) and behavioural inhibition system (BIS). The BAS scale is further divided into 3 subscales: Drive, which assesses individual’s inclination to pursue desired goals; fun seeking, related to the desire for new rewards and impulsive drive towards potential rewards; and reward responsiveness. The BAS is thought to be responsible for responding to incentives with positive affect and increased motivation. On the other hand, the BIS triggers experiences of anxiety, fear and negative affect in response to threatening stimuli. Higher scores on both subscales are associated with higher sensitivity of the given system. Based on the previous literature^41,42^ we expected autistic traits to be associated with higher BIS scores and lower BAS reward responsiveness subscale scores.

The LSAS-SR is a self-report scale assessing anxiety related to experiencing everyday social situations; higher scores indicate stronger anxiety. The motivation for employing the LSAS-SR in the study were findings of modulatory effect of social anxiety on social reward processing^43^ and increased levels of social anxiety among population with autism^19^. We expected to see a positive correlation between the AQ and the LSAS-SR scores.

Additionally, participants answered the following debriefing questions: *How motivated were you to perform well in the experiment?* (general motivation); *Was the incentive type important to you?* (importance of condition); *Which reward was the most motivating one for you?* (motivational value of cues); *How rewarding did you find the feedback pictures?* (rewarding value of feedback).

## Data analysis

All data analyses were performed using R ver. 3.4.3^44^. The significance level for all the tests was set to 0.05.

### Brain responses

To assess potential effects of autistic traits and reward type on brain responses we utilised multiple regression analyses with mixed effects through the lmerTest package ver. 2.0.36^45^. Random intercepts for subjects were used in all multiple regression models based on improvement of Akaike’s Information Criterion^46^ upon their addition to an intercept-only model. Random effects allow analysing hierarchical data while accounting for non-independence of the measures^47^. Assumptions for multiple regression were checked for all models (normality, linearity, multicollinearity, homoscedasticity). Marginal and conditional R^2^ were calculated as measures of goodness of fit for mixed models^48^, in which marginal R^2^ (R^2^_m_) reflects variance explained by fixed factors, and conditional R^2^ (R^2^_c_) - variance explained by the entire model. The *p*-values were computed via Wald-statistics approximation (treating t as Wald z). To estimate a main effect of the incentive type, which is a multilevel categorical predictor, we administered an analysis of variance with Satterthwaite approximation for degrees of freedom and type II sums of squares on the regression models^45^. Here we report only the significant effects, with Bonferroni-corrections where applicable. The complete regression tables including two contrasts (SM and M) are reported in Supplementary Material. Since there is no established way of calculating standardised effect sizes for individual model terms in linear mixed models due to the way the variance is partitioned^49^, see Supplementary Material for all unstandardized slope estimates, which are the essential effect size statistics^50^.

The temporal windows and regions of interest (ROI) for the CNV in response to cues and in the pre-feedback waiting period, and for the P3 in response to feedback, were chosen based on prior research and visual inspection of grand averages. The resulting time windows for anticipation phases (cue and pre-feedback) were the last 500 ms of each phase. For the cue this was calculated as 1000–1500 ms after cue onset (with −100 – 0 ms baseline). For the jittered pre-feedback the anticipation phase was time-locked to the onset of the feedback and defined as −500 – 0 ms (with −100 – 0 ms baseline locked to the onset of the pre-feedback fixation cross). The feedback P3 was identified from 300 to 500 ms after feedback onset. Mean amplitudes for these time windows were calculated from 9 electrodes for the CNV (CPz, CP1, CP2, Pz, P1, P2, POz, PO3, PO4) and from 9 electrodes for the P3 (Cz, C1, C2, CPz, CP1, CP2, Pz, P1, P2).

To explore the influence of autistic traits on brain responses we built multiple regression models with AQ score (continuous measure) and incentive type (S, M, SM) as the main predictors. To compare anticipatory responses across time, a model with additional phase (cue, pre-feedback) as a predictor was built. For the analysis of reward reception, AQ score, incentive type and outcome (reward, no-reward) were included. We controlled for social anxiety by including the LSAS-SR score in all the models.

### Questionnaires and debriefing questions

The AQ, LSAS-SR and BIS/BAS scores were used as continuous measures of autistic traits, social anxiety, and approach/avoidance behaviour. All questionnaire scores were centred before including them in the models. Correlations between the questionnaires were computed using Pearson’s rank correlation coefficients.

## Results

### ERPs

#### Anticipatory CNV amplitudes – cue and pre-feedback

**Figure 2:**
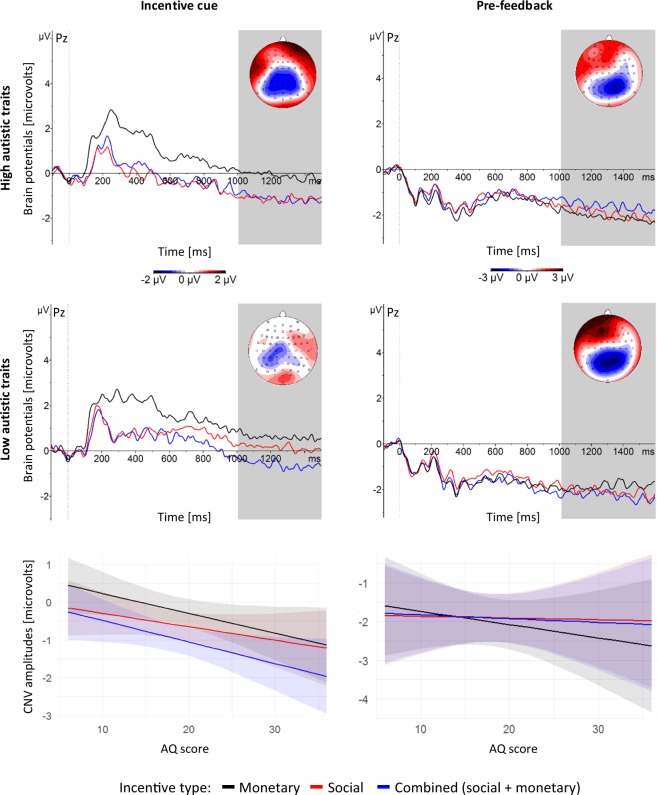
Top two rows: brain responses at the Pz electrode time-locked to the onset of the incentive cue (left side) and the pre-feedback waiting period (right side), averaged over participants with high- (top row) and low (middle row) autistic traits (based on a median-split, Mdn = 17). Topographic maps show scalp distributions at indicated time intervals. Note that pre-feedback CNV amplitudes are displayed here as locked to the onset of the waiting period, but were quantified for analyses relative to feedback onset, due to the variable lengths of the waiting period. Bottom row: mean predicted CNV amplitudes in the last 500 ms of the incentive cue presentation (left side) and the last 500 ms of the pre-feedback waiting period (right side). The shadowed bands indicate confidence intervals.

We analysed attenuated anticipatory brain responses in relation to autistic traits by conducting a standard multiple regression analysis with the outcome variable of CNV amplitudes and predictor variables of autistic traits (AQ scores) and incentive type (S, M, SM). Figure 2 visualises the CNV values predicted by the models for both anticipation phases (early and late), with a median-split (Mdn = 17) of the sample into groups with high- and low autistic traits. The median-split was only used in the plots; all analyses were performed on continuous AQ score.

In the incentive cue phase, analysis of variance with Satterthwaite approximation for degrees of freedom and type II sums of squares on the regression model yielded a statistically significant effect of AQ, *F*(1,51) = 4.81, *p* = 0.03, and incentive type, *F*(2,102) = 8.31, *p* < 0.001. In the regression model, AQ predicted more negative CNV amplitudes with larger effects in SM (*β* = −0.06), than M (*β* = −0.05), than S (*β* = −0.04), but none of these contrasts survived corrections for multiple comparisons (corrected *p*s = 0.1, 0.14, 0.5, respectively). Pair-wise contrasts of incentive types were statistically significant after correction only for SM vs. M (*β* = −0.12, *p* < 0.001), with the largest amplitudes for SM, followed by S and M.

In the pre-feedback phase the analysis of variance on the regression model showed statically significant effect only of the covariate, LSAS-SR, *F*(1,51) = 5.02, *p* = 0.03 (in regression *β* = 0.03 for all contrasts, corrected *p*s = 0.75).

Together, these results suggest that autistic traits are related to enhanced CNV amplitudes in response to an incentive cue indicating a future reward type. Unrelated to AQ, participants showed the largest responses to anticipation of the combined SM rewards. These effects disappear directly before reception of the reward (in the pre-feedback phase). Here, social anxiety seems to be inversely related to the CNV amplitudes.

#### CNV responses across anticipation phases

To explore the CNV changes across anticipation phases, we built a model with the CNV as the outcome variable, AQ, incentive type and phase (cue, pre-feedback) as predictors, and LSAS-SR as a covariate.

**Figure 3:**
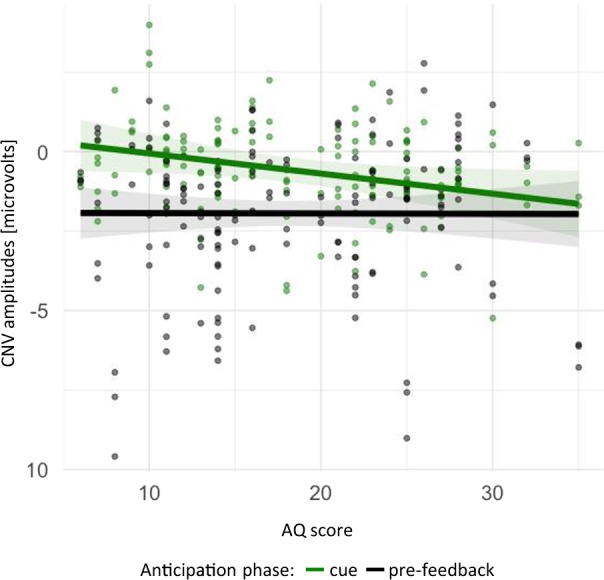
Mean predicted CNV amplitudes for AQ scores in both anticipation phases (negative CNVs indicating enhanced anticipation). The shadowed bands indicate confidence intervals.

Analysis of variance on this model yielded an interaction effect between autistic traits and phase, *F*(1,255) = 7.57, *p* = 0.006. The interaction between phase and AQ is presented in Fig. 3. Autistic traits did not modulate the CNV in the pre-feedback phase. However, in response to incentive cues, autistic traits did indeed modulate the CNV, with higher trait levels eliciting larger amplitudes. This suggests that autistic traits play a greater role in early anticipation than in late reward anticipation.

The analysis of variance revealed also a main effect of phase, *F*(1,255) = 66.94, *p* < 0.001 (in the regression model the contrast of cue vs. pre-feedback also yielded a main effect of phase, with more negative CNV amplitudes in the pre-feedback, with the largest effect in M (*β* = 1.8, smaller in S (*β* = 1.3), and the smallest in SM (*β* = 0.93); for all contrasts corrected *p*s < 0.003), and LSAS-SR, *F*(1,51) = 4.44, *p* = 0.04 (in regression, *β*s = 0.02 across contrasts; corrected *p*s = 0.11). Since LSAS-SR was utilised as a covariate and phase showed a significant interaction effect with another predictor, these two main effects were no longer investigated.

#### The P3 amplitude in response to feedback

To explore the brain responses during feedback processing, a multiple regression model was built with P3 amplitudes as the dependent variable, AQ, incentive type and outcome (reward, no reward) as predictors, and LSAS-SR as a covariate.

Analysis of variance of this model revealed a significant main effect of reward type, *F*(2,255) = 44.21, *p* < 0.001, and regression results confirmed statistically significant differences for S vs. M (*β* = 1.25) and S vs. SM (*β* = 1.3; for both contrasts corrected *p*s < 0.001). P3 responses to SM and M were statistically indistinct (*β* = −0.05, corrected *p* > 1). The analysis of variance also revealed a main effect of outcome (reward vs. no-reward), *F*(1,255) = 10.2, *p* = 0.002, with larger P3 amplitudes for reward outcomes (*β*s = 0.39–0.43 across contrasts, all corrected *p*s > = 0.18).

**Figure 4:**
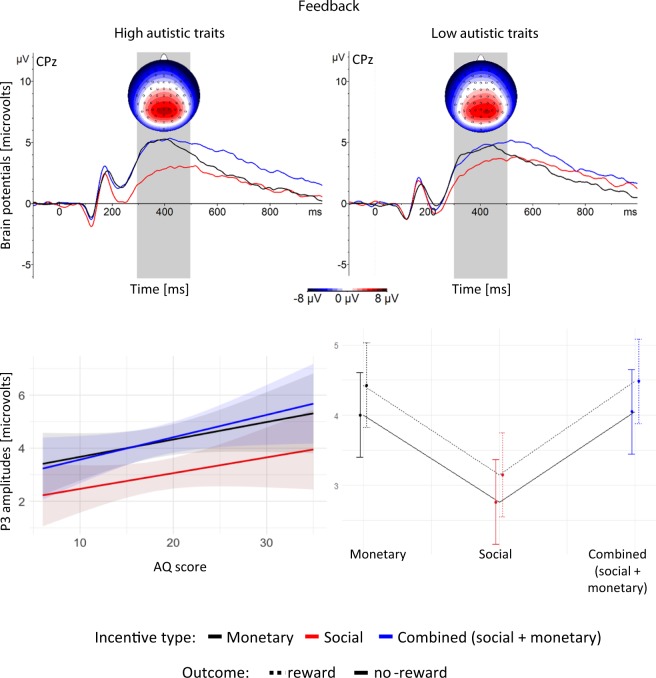
Top row: brain responses at electrode CPz, time-locked to the onset of feedback, averaged over participants with high- (left panel) and low (right panel) autistic traits (based on a median-split, Mdn = 17). Topographic maps show the scalp distributions across conditions at indicated time intervals. Bottom row: mean predicted P3 amplitudes for 300–500 ms after feedback onset across the AQ scores (left panel) and across incentive type and outcome (right panel). The shadowed bands indicate confidence intervals and the error bars show standard error.

Figure 4 shows the significant effects revealed in the analysis of variance administered to the model. Rewards elicited larger P3 amplitudes than no rewards. Both M and SM triggered larger responses than S.

### Behavioural data

#### Questionnaires

We found statistically significant correlations between AQ scores and LSAS-SR (*r* = 0.38, *p* = 0.006), BIS scale (*r* = 0.54, *p* < 0.001), BAS reward responsiveness scale (*r* = −0.29, *p* = 0.04), and BAS fun seeking scale (*r* = −0.3, *p* = 0.03). The correlation between AQ and BAS drive scores showed a trend effect (*r* = −0.27, *p* = 0.06). Together, these results point at the relation of higher autistic traits and increased social anxiety, higher sensitivity of the inhibition system, and weaker responsiveness to anticipation or occurrence of rewards. Altogether, these results suggest that individuals with high autistic traits are motivated stronger by avoidance of punishment than by drive to rewards.

#### Debriefing questions

Participants with high and low autistic traits did not differ in their general self-reported motivation during the experiment, *t*(48.1) = −1.35, *p* = 0.18, nor in the ratings of motivational power of incentive cues, *χ*^2^(2) = 2.01, *p* = 0.37, or in perceived importance of incentive types, *χ*^2^(1) = 0, *p* = 1. Autistic traits were also unrelated to subjective reward values of the social (*r* = −0.13, *p* = 0.36), or non-social feedback pictures (*r* = 0.07, *p* = 0.61). Altogether, autistic traits did not influence any self-reported behavioural measures.

## Discussion

This study used a modified version of a cued incentive delay task with socially relevant faces to explore brain sensitivity to social, monetary and combined social-monetary rewards in neurotypical participants with varying levels of autistic traits. We observed enhanced early anticipation of rewards in high autistic traits when triggered by symbolic, incentive cues representing possible outcomes of the conditions, as reflected in the CNV amplitudes. The brain responses were largest when anticipating the combined (social and monetary) rewards, independently of autistic traits. All these effects disappeared in late anticipation, i.e., directly before the reception of rewarding/non-rewarding feedback. A secondary analysis revealed an interaction effect of AQ scores and anticipation phase, suggesting that autistic traits were associated with increased anticipation during the early phase (triggered by a symbolic incentive cue), but did not modulate the responses in the late anticipation phase (directly before reward reception). Appreciation of received rewards reflected in higher P3 amplitudes was the largest for monetary and combined outcomes across levels of autistic traits.

Contrary to our hypotheses, our data do not support the predictions of the social motivation account, as we did not observe any interactions of autistic traits and reward types. However, a recent systematic review^51^ examined 27 studies out of which 15 did and 12 did not support the social motivation hypothesis, which suggests that our results are not incidental. The heterogeneity of results reported so far calls for more research to examine the underlying mechanisms of social and non-social reward processing in autism and autistic traits, which are not yet well explained. Moreover, our results have to be considered in the light of the experimental design: One of its goals was to increase ecological validity of social stimuli by introducing pictures of the main experimenter, who was a relevant interaction partner, whereas most other studies used faces of strangers^6–8,24,52,53^. Our choice was based on reports of reduced activity of the fusiform face area in individuals on autism spectrum in response to pictures of strangers, but not familiar faces^18^ and normalised pupillary responses to familiar, but not to strangers’ faces^54^. Atypical processing of faces has also been observed across autistic traits^55^. The CNV amplitude in the early anticipation was indeed modulated by the incentive type, with incentives with social components reaching larger amplitudes than the solely monetary one. Therefore, it seems likely that presenting familiar (all participants recognised the person in the stimuli pictures as the experimenter prior to the task) and socially relevant (the experimenter and the participants shared a social context, in which the experimenter was an important interaction partner) stimuli might have normalised responsiveness to social reward in participants with higher levels of autistic traits. Even though, it is important to keep in mind that a single smiling face on a computer screen does not mimic natural social interactions, as faces in such situations are always seen in a complex context. However, by using a familiar and relevant face we aimed to increase the ecological validity while maintaining both high experimental control and comparability to the large body of existing studies using smiling faces as rewards. So far, two studies directly compared familiar vs. unfamiliar faces in reward-based paradigms. One of them^35^ used faces as incidental rewards (co-occurring with food rewards), which made them redundant for retrieving the feedback valence information and does not allow independent assessment. Since processing of the faces was not task-relevant, the results of this study cannot be compared directly to our design, in which processing of the faces was crucial for retrieving both rewarding and informative values of the feedback. In the second study^11^ children with ASC benefited more than the control group from both familiar and unfamiliar social (as well as non-social) rewards, as measured with reaction times and false alarms rates. This suggests increased reward responsiveness in the ASC group, which is in line with our finding of enhanced reward anticipation in higher autistic traits. However, to our knowledge no study to date has investigated familiarity effects across autistic traits. It is possible that brain responses to irrelevant (unfamiliar) social stimuli differ between individuals with high and low autistic traits when such stimuli convey informative feedback and reward. Although this should be addressed in a study specifically targeting this question, this interpretation of our results suggests that the relevance of social stimuli might modulate social motivation. Alternatively, interaction effects of incentive types and autistic traits could have been unobserved in our data because such modulations might not manifest in subclinical levels of autistic traits. However, previous studies have documented the interaction effects in neurotypical samples varying in levels of autistic traits before^16,17^ and have further shown associations for a range of other cognitive and emotional characteristics that are relevant to autism^55–58^, which makes this an unlikely explanation for our pattern of results. Therefore, we favour the interpretation that the use of socially relevant faces in our study normalised the reward responsiveness to social rewards across autistic traits.

We observed that higher autistic traits were associated with stronger CNV responses to incentive cues of all reward types. The CNV is believed to reflect expectation of an upcoming event, and to be modulated by motivation and anticipation of affective stimuli^32^. Our results suggest that higher autistic traits were linked to larger anticipatory activation reflecting forming of reward representations in response to incentive cues. Though autism has primarily been linked to *reduced* reward anticipation^8,24,25^, some findings have been reported that are in line with our results. One study^11^ observed greater behavioural responsiveness of autistic than control children to reward contingencies, given their baseline at a no-reward condition. Other studies observed larger anticipatory ERP amplitudes in children with autism than in a control group when expecting positive feedback (and non-social reward)^30^, and increased activation of multiple brain areas (i.e., nucleus accumbens and hippocampus to monetary incentives; amygdala and insular cortex to social incentives) in ASC during reward processing^15^. An alternative explanation of our results, which do not match the majority of findings on the topic, could be that larger CNV amplitudes represent arousal for the upcoming task performance, rather than reward anticipation^59^. However, observed differences between reward types, with the strongest responses to the incentive of combined (social and monetary) reward, make this unlikely. Such pattern rather suggests an additive motivating value of the social and monetary reward incentives, and adds to the interpretation of the CNV amplitudes as an indicator of reward anticipation.

Neither AQ nor reward type effects manifested in the pre-feedback waiting period directly preceding reward reception, despite visible anticipatory brain responses occurring in this time-window in the form of the CNV. The secondary analysis of anticipation over time yielded an interaction effect of AQ scores and anticipation phase, revealing that autistic traits affected processing in the early anticipation stronger than in the late one. Further, reward anticipation as indexed by the CNV was overall stronger in the late phase, albeit not influenced by either reward type or autistic traits.

To our knowledge, this study is the first one to investigate the early phase of reward anticipation in the context of autism. We used abstract representations of possible future rewards as incentive cues, triggering early anticipation of rewards. Oftentimes studies used the same stimulus (typically a smiling face) as both an incentive cue, which communicates a future possible reward, and as a feedback stimulus serving as the obtained reward^8,16^. This confounds reward anticipation and reward reception, making it impossible to interpret responses to cues as solely reflecting reward anticipation. This is especially important in paradigms comparing processing of social versus monetary rewards. Symbolic monetary outcomes (e.g. a picture of a coin) usually indicate future reception of a tangible reward (cash reimbursement). Social feedback typically consists of a smiling face, which is in itself a transient, immediate reward. Using a smiling face as an incentive cue is supposed to only indicate a future rewarding smiling face, which is later delivered with the same stimulus. Hence, in such designs reward anticipation cannot be interpreted, as it indeed includes reward reception. To extract the anticipation unaffected by prematurely delivered reward, we utilised symbolic representations of the rewards. Time-locking brain potentials to the onset of symbolic incentive cues in this study revealed modulatory effects of autistic traits (larger responses in high traits) and reward types (the largest responses for the combined social and monetary incentives). This suggests that the incentive cues indeed elicited representations and anticipation of future rewards, as reflected in the occurrence of an anticipatory CNV component.

This sensitivity to cue – reward associations in higher autistic traits regardless of reward type was unexpected and stands at odds with accounts proposing impaired forming of stimulus-reward associations in autism resulting in decreased reward anticipation^60^. Since reward sensitivity is essential for conditioning processes and reinforcing appropriate behaviours (leading, in turn, to successful social interactions), it is crucial to understand the reasons for mixed results reported in the literature. One explanation could be that the atypical pre-feedback processing in participants with high autistic traits found in other studies^16^ does not result from an inability to build stimulus-outcome associations, but rather reflect differences in the time course of reward anticipation between high and low autistic traits. Considering the CNV amplitudes in the early and late stages of reward anticipation in our paradigm, high autistic traits seem to be linked to more stable responses over time – not diminished directly before reward reception, but rather enhanced when triggered by incentive cues. In contrast, low autistic traits were associated with increasing anticipation over time – less anticipation than for high autistic traits in response to incentive cues, but increasing shortly before receiving the feedback and rewards, resulting in comparable reward anticipation for high and low autistic traits directly before reward reception. It is not clear what drives this difference in trajectory between low and high traits. Perhaps forming representations of rewards based on abstract cues is facilitated in higher levels of autistic traits. Possibly other inter-individual features in individuals with higher autistic traits in our sample, like anxiety or increased sensitivity to punishment (reflected in statistically significant correlations between AQ and LSAS-SR / BIS scores, respectively), modulated the processing shortly before outcome reception, refraining further excitation of the reward system. Altogether, our findings of increased responses in early but not late anticipation of rewards associated with high autistic traits demonstrate that targeting neural mechanisms underlying early responses to abstract representations of rewards offer new insights into reward sensitivity in autism.

We observed that the P3 component was elicited in response to feedback stimuli. The P3 amplitudes were larger for the monetary and the combined (social + monetary) conditions, than for the social condition. Since the social and combined rewards consisted of the same stimuli, the different brain responses to them imply that participants were aware of the reward type indicated by the preceding symbolic incentive cues. This provides further evidence that incentive cues successfully elicited reward representations. There is no evidence for social and monetary rewards being additive, as both the combined and the monetary-only outcomes elicited similar responses: participants seem to have appreciated the monetary component when both accompanied and unaccompanied by social appreciation (preference for monetary rewards has been observed in neurotypicals before^8,61^). Since the P3 has been previously shown sensitive to reward magnitude and valence^33,62^, the data suggest a preference for tangible rewards over transient ones, at least in experimental settings.

Interestingly, differences across autistic traits and reward types were only visible in neural responses. Self-reported motivational and rewarding values of incentive cues and rewards did not differ between participants with low and high autistic traits. Hence, the brain responses in this study add to the growing body of literature stressing sensitivity of ERPs to cognitive and affective processes which may not manifest in behaviour^8,16^.

Our study contributes to autism research by investigating autistic traits in a population-based approach, and to the existing literature on reward responsiveness by proposing a novel insight into dynamics of anticipation over time (across paradigm phases). By using pictures of a socially relevant interaction partner as reward stimuli we aimed to provide an experimental context better resembling natural social situations. Our finding of enhanced early but not late reward anticipation in high autistic traits offers a new insight into the dynamics of reward processing in the context of the autism spectrum. It also demands a more refined interpretation of the social motivation account and its predictions about aberrant (multi)dimensional responsiveness to rewards in autism.

Moreover, this study offers a population-based view, in which neurotypical participants vary in levels of autistic traits and individuals diagnosed with autism would represent the extreme levels of these traits. Our results of autistic traits modulating reward anticipation stress the need for a careful control of neurotypical groups serving as controls for autism spectrum subjects in psychological research. Apparent heterogeneity of sub-clinical (control) populations in terms of reward responsiveness may contribute to explaining mixed results in the literature exploring differences in reward sensitivity between individuals with and without autism.

Finally, targeting various stages of reward processing (early and late reward anticipation, reward reception) allows a more fine-grained characterisation of functional and dysfunctional reward processing. Since functional disconnection of reward processing phases has been observed in other groups with reward processing dysfunctions (addicts^63^), it can also have implications for autism. Identifying atypical stages of reward responsiveness in autism can facilitate personalised therapies by selectively targeting the vulnerable sub-processes, which in turn are likely to reinforce desired therapy effects^64^.

## Supplementary information

Supplementary material.

## Data Availability

The datasets generated during the current study are not publicly available due to lack of corresponding explicit agreement phrase in used consent forms, but are available from the corresponding author upon request.
