# Supplementary material for: Autistic Traits Affect Reward Anticipation but not Reception
Source: Sci Rep. 2020 May 21;10:8396. doi: 10.1038/s41598-020-65345-x (PMC7242422; doi:10.1038/s41598-020-65345-x)
Supplement: Supplementary file 1 — Supplementary material. [file 41598_2020_65345_MOESM1_ESM.pdf]

Supplementary material to

Autistic Traits affect Reward Anticipation but not Reception

Magdalena Matyjek, Mareike Bayer, Isabel Dziobek

Note:

CI = Confidence Intervals

σ^2 = within group variance

τ_{00} = between group variance

ICC = Interclass Correlation (the ratio of the between-cluster variance to the total variance)

AIC $_{\text{model/null model}}$ = Akaike Information Criterion of the model in discussion or the null model (containing only intercept as a predictor)

P-values were computed via Wald-statistics approximation (treating t as Wald z), uncorrected.

Supplementary material - table 1 Regression results for the CNV in response to cues

	CUE: CNV									
Predictors	*Estimates*	*SE*	*CI (0.95)*	*t*	*p*	*Estimates*	*SE*	*CI (0.95)*	*t*	*p*
**Intercept**	-0.98	0.18	-1.33 – -0.62	-5.36	**<0.001**	-0.22	0.18	-0.58 – 0.14	-1.21	0.225
**LSAS-SR**	0.01	0.01	-0.01 – 0.02	0.76	0.446	0.01	0.01	-0.01 – 0.02	0.76	0.446
**AQ**	-0.06	0.03	-0.11 – -0.00	-2.15	**0.032**	-0.05	0.03	-0.10 – -0.00	-1.98	**0.048**
**SM - M**	0.75	0.19	0.39 – 1.12	4.08	**<0.001**					
**SM - S**	0.38	0.19	0.01 – 0.74	2.03	**0.042**					
**M - S**						-0.38	0.19	-0.74 – -0.02	-2.05	**0.040**
**AQ : SM - M**	0.00	0.03	-0.05 – 0.05	0.17	0.864					
**AQ : SM - S**	0.02	0.03	-0.03 – 0.07	0.84	0.402					
**AQ : M - S**						0.02	0.03	-0.03 – 0.07	0.67	0.505

**Random Effects**		**Model Information**	
σ^2	0.87	Observations	153
$\tau_{00\ code}$	0.82	Marginal R^2 / Conditional R^2	0.108 / 0.540
ICC_{code}	0.48	AIC_{model} / $AIC_{null\ model}$	499.513 / 535.919

Supplementary material - table 2 Regression results for the CNV in the pre-feedback phase

	PRE-FEEDBACK: CNV									
Predictors	*Estimates*	*SE*	*CI (0.95)*	*t*	*p*	*Estimates*	*SE*	*CI (0.95)*	*t*	*p*
**Intercept**	-1.91	0.31	-2.52 – -1.30	-6.15	**<0.001**	-2.03	0.31	-2.64 – -1.42	-6.55	**<0.001**
**LSAS-SR**	0.03	0.01	0.00 – 0.06	2.24	**0.025**	0.03	0.01	0.00 – 0.06	2.24	**0.025**
**AQ**	-0.01	0.05	-0.10 – 0.08	-0.21	0.831	-0.03	0.05	-0.12 – 0.06	-0.75	0.455
**SM - M**	-0.12	0.19	-0.50 – 0.26	-0.62	0.532					
**SM - S**	0.00	0.19	-0.38 – 0.39	0.03	0.980					
**M - S**						0.13	0.19	-0.25 – 0.51	0.65	0.516
**AQ : SM - M**	-0.02	0.03	-0.08 – 0.03	-0.91	0.361					
**AQ : SM - S**	0.01	0.03	-0.05 – 0.06	0.20	0.841					
**AQ : M - S**						0.03	0.03	-0.02 – 0.08	1.11	0.265

**Random Effects**		**Model Information**	
σ^2	0.96	Observations	153
$\tau_{00\ code}$	3.95	Marginal R^2 / Conditional R^2	0.085 / 0.821
ICC_{code}	0.80	AIC_{model} / $AIC_{null\ model}$	587.013 / 695.147

Supplementary material - table 3 Regression results for the CNV in two phases – cue and pre-feedback

	CUE and PRE-FEEDBACK: CNV									
Predictors	*Estimates*	*SE*	*CI (0.95)*	*t*	*p*	*Estimates*	*SE*	*CI (0.95)*	*t*	*p*
**Intercept**	-0.98	0.26	-1.48 – -0.48	-3.81	**<0.001**	-0.23	0.26	-0.73 – 0.28	-0.88	0.380
**LSAS-SR**	0.02	0.01	0.00 – 0.04	2.11	**0.035**	0.02	0.01	0.00 – 0.04	2.11	**0.035**
**AQ**	-0.07	0.04	-0.14 – 0.00	-1.95	0.052	-0.07	0.04	-0.14 – 0.00	-1.83	0.067
**SM - M**	0.75	0.28	0.20 – 1.31	2.66	**0.008**					
**SM - S**	0.38	0.28	-0.18 – 0.93	1.32	0.186					
**M - S**						-0.38	0.28	-0.94 – 0.18	-1.33	0.182
**cue - prefeedback**	-0.93	0.28	-1.48 – -0.37	-3.26	**0.001**	-1.80	0.28	-2.36 – -1.25	-6.34	**<0.001**
**AQ: SM - M**	0.00	0.04	-0.07 – 0.08	0.11	0.911					
**AQ : SM - S**	0.02	0.04	-0.06 – 0.10	0.55	0.585					
**AQ : M - S**						0.02	0.04	-0.06 – 0.09	0.43	0.664
**AQ : cue - prefeedback**	0.08	0.04	0.00 – 0.15	1.97	**0.049**	0.05	0.04	-0.03 – 0.13	1.23	0.217
**SM - M : cue - prefeedback**	-0.88	0.40	-1.66 – -0.09	-2.18	**0.029**					
**SM - S : cue - prefeedback**	-0.37	0.40	-1.16 – 0.42	-0.92	0.356					
**M – S : cue – prefeedback**						0.51	0.40	-0.28 – 1.29	1.26	0.208
**AQ : SM - M : cue - prefeedback**	-0.03	0.06	-0.14 – 0.08	-0.52	0.603					
**AQ : SM - S : cue - prefeedback**	-0.02	0.06	-0.12 – 0.09	-0.29	0.773					
**AQ : M - S : cue - prefeedback**						0.01	0.06	-0.10 – 0.12	0.23	0.817

**Random Effects**		**Model Information**	
σ^2	2.05	Observations	306
$\tau_{00\ code}$	1.32	Marginal R^2 / Conditional R^2	0.176 / 0.498
ICC_{code}	0.39	AIC_{model} / $AIC_{null\ model}$	1199.097 / 1303.481

Supplementary material - table 4 Regression results for the P3 in response to outcomes

	FEEDBACK: P3									
Predictors	*Estimates*	*SE*	*CI (0.95)*	*t*	*p*	*Estimates*	*SE*	*CI (0.95)*	*t*	*p*
**Intercept**	4.08	0.31	3.47 – 4.68	13.26	**<0.001**	4.03	0.31	3.42 – 4.63	13.10	**<0.001**
**LSAS-SR**	-0.01	0.01	-0.04 – 0.01	-0.88	0.378	-0.01	0.01	-0.04 – 0.01	-0.88	0.378
**AQ**	0.09	0.05	0.00 – 0.18	2.00	**0.046**	0.07	0.05	-0.01 – 0.16	1.66	0.097
**SM - M**	-0.05	0.22	-0.49 – 0.39	-0.22	0.823					
**SM - S**	-1.30	0.22	-1.74 – -0.86	-5.79	**<0.001**					
**M - S**						-1.25	0.22	-1.69 – -0.81	-5.56	**<0.001**
**no-reward – reward**	0.43	0.22	-0.01 – 0.87	1.93	0.054	0.42	0.22	-0.02 – 0.86	1.87	0.062
**AQ: SM - M**	-0.02	0.03	-0.08 – 0.05	-0.49	0.622					
**AQ : SM - S**	-0.03	0.03	-0.09 – 0.03	-1.09	0.276					
**AQ : M - S**						-0.02	0.03	-0.08 – 0.04	-0.60	0.551
**AQ : no-reward – reward**	-0.01	0.03	-0.07 – 0.05	-0.38	0.701	-0.02	0.03	-0.08 – 0.04	-0.60	0.548
**SM - M : no-reward – reward**	-0.01	0.32	-0.63 – 0.61	-0.04	0.968					
**SM - S : no-reward – reward**	-0.04	0.32	-0.66 – 0.58	-0.13	0.894					
**M – S : no-reward – reward**						-0.03	0.32	-0.65 – 0.59	-0.09	0.927
**AQ : SM - M : no-reward – reward**	-0.01	0.04	-0.09 – 0.08	-0.15	0.878					
**AQ : SM - S : no-reward – reward**	0.02	0.04	-0.07 – 0.10	0.40	0.688					
**AQ : M - S : no-reward – reward**						0.02	0.04	-0.06 – 0.11	0.56	0.579

**Random Effects**		**Model Information**	
σ^2	1.28	Observations	306
$\tau_{00\ code}$	3.53	Marginal R^2 / Conditional R^2	0.118 / 0.766
ICC_{code}	0.73	AIC_{model} / $AIC_{null\ model}$	1119.680 / 1391.224
